# Open Science Practices in Integrated Assessment Models

**DOI:** 10.12688/openreseurope.18824.1

**Published:** 2025-01-20

**Authors:** Clàudia Rodés-Bachs, Jon Sampedro, Natasha Frilingou, Francesco Gardumi, Camilla Lo Giudice

**Affiliations:** 1 Basque Center for Climate Change, Leioa, Basque Country, Spain; 2 Energy Policy Unit, School of Electrical and Computer Engineering, National Technical University of Athens, Iroon Politechniou 9, 157 80 Athens, Greece; 3 KTH Royal Institute of Technology, Brinellvägen 68, 114 28 Stockholm, Stockholm County, Sweden

**Keywords:** open science, integrated assessment models, protocol, transparency, FAIR, TRUST, open-source, open data

## Abstract

**Background:**

Open science emphasizes the free and accessible dissemination of scholarly outputs to a wide audience, including scientists, stakeholders, and the general public. Its core principle is the open sharing of knowledge to enable reuse, replication, and uphold research integrity.

**Methods:**

Using insights from a survey designed to explore the open science practices within integrated assessment modeling (IAM) teams, as well as the challenges and barriers they face, we propose an open science protocol tailored specifically to the needs of IAMs.

**Results:**

The proposed protocol improves the transparency, accessibility, reliability, reusability, and interoperability of IAM models and results. Grounded in the findability, accessibility, interoperability, and reusability (FAIR) and transparency, responsibility, user focus, sustainability, and technology (TRUST) principles, it supports the transformation of models’ outputs into real-world applications.

**Conclusions:**

By fostering enhanced trust and engagement from policymakers, this protocol supports the broader adoption of open science in IAMs. It is complemented by a checklist and includes recommendations for open-source platforms and tools, simplifying workflows and minimizing the need for specialized expertise.

## Plain language summary

Open science encourages the free sharing of research and data with scientists, policymakers, and the general public, aiming to make knowledge accessible for reuse and further development. To improve the reliability and widespread adoption of these practices, many scientific communities have developed tailored protocols and standardized methods. Integrated assessment models (IAMs), which provide quantitative insights for global environmental change and sustainable development, and feed into major scientific assessments, must be transparent and open to support informed decision-making. Based on a survey of open science practices carried out by different IAM teams, we developed a protocol to address key challenges and overcome existing barriers in the use of open science practices in integrated assessment modelling. This protocol enhances the transparency, accessibility, and reliability of IAMs and their results, fostering greater trust among policymakers and helping to transform model results into real-world applications.

## State of the art in open science practices

Open science is a concept that aims to make scientific research and data available to scientists, stakeholders, and the general public. The main principle of open science is based on the idea that scientific knowledge should be freely available to everyone and should be easily accessible for reuse, replication, and further development ^1^. It rests on a set of core values ^2, 3^, including transparency, reproducibility, accessibility, and interdisciplinarity, to ensure that scientific research is conducted in an open and transparent manner, and that the resulting data, methodologies, and findings are made available to the broader scientific community and non-expert audiences ^4^. By making scientific research and data available to everyone, open science can help to promote scientific progress and drive innovation ^5^.

The concept of open science has gained momentum in recent years, with a significant number of researchers, policymakers, and funding agencies recognizing the importance of openness in scientific research ^6– 8^. Researchers from a diverse range of fields strive to remove barriers to information exchange and encourage the academic community to embrace open science procedures ^5^. Numerous initiatives and policies also drive the adoption of open science, such as the European Union’s Horizon 2020 program and the US National Institutes of Health, which have implemented open-access mandates ^9, 10^. Meanwhile, the European Commission created the EU’s Open Science Policy Platform to provide high-level guidance on the development of its Open Science Policy ^11^.

There is an increasing number of resources and platforms available to support open science, including open-access journals, indexed and browsable in the Directory of Open Access Journals ^1^; open data repositories, such as the World Bank open data ^2^ or the World Health Organisation data ^3^; software for open-source computing and development, for instance, Unix ^4^ and Git ^5^; and projects to promote open data-driven science, such as the European Open Science Cloud ^6^ and the Open Energy Modelling Initiative ^7^; all of which can help to ensure that clear and accessible research methods are used.

As open data gains prominence, the FAIR principles have emerged as a measurable guideline for assessing the Findability, Accessibility, Interoperability, and Reusability of scientific data ^2^. These principles, adopted by institutions like the European Commission, promote data exchange and usage, enabling researchers to build upon existing knowledge. However, the FAIR principles face limitations, including a focus on individual practices and short-term data management ^12, 13^. To address these potential barriers, the TRUST principles, based on the pillars of Transparency, Responsibility, User focus, Sustainability, and Technology, were introduced in 2020, ensuring digital repository trustworthiness and long-term data preservation ^3^.

The benefits of open science are wide-ranging. First, it improves research efficiency and reduces total costs through multiple factors such as labor cost savings, productivity improvements, access cost savings, or duplication avoidance ^14^. Importantly, it facilitates interdisciplinarity by using new products, services, companies, and collaborations, which would have been less likely to occur in closed environments ^14, 15^. Likewise, open science practices increase public engagement by making research more accessible and understandable to non-experts, which expands reliability and funding opportunities ^14, 16, 17^. A representative example of the benefits of open science practices is the scientific research during the COVID-19 pandemic. In order to boost scientific collaboration to fight the pandemic, several publishers and researchers sped up their adoption of open science practices ^18, 19^. Reviewing processes were accelerated and preprints were shorter and more quickly available ^20^. In the same way, data —especially COVID-19 related— were shared among different institutions and countries, which enhanced the transparency and cooperation between scientists. While this new research paradigm and the new sharing and publication rhythm raised concerns about the potential impact on the quality of research output ^18^, it also encouraged the development of additional guidelines and specific protocols to guarantee the successful deployment of open science practices, which have been maintained in the post-pandemic era.

## Open science practices in IAMs

Open science is gaining momentum across various scientific disciplines ^21– 24^. The integrated assessment modeling community has been applying its principles, with the aim to improve the transparency, accessibility, and understanding of integrated assessment models (IAMs) generated scenarios ^25, 26^. IAMs are essential tools for global scenario analysis and for exploring the systemwide impacts of climate policies ^27^. For instance, their outcomes and results have been used in the latest Intergovernmental Panel on Climate Change (IPCC) report (AR6; ^28^), which required all contributors to homogenize their data to enable comparisons and ensure full transparency ^29^.

Several well-established IAMs are freely available as open-source projects in their respective repositories, such as GCAM ^8^, MESSAGEix ^9^, MUSE ^10^, REMIND ^11^, and WITCH ^12^. Moreover, efforts are underway to transition models to open access formats, such as OMNIA and OPEN-PROM, which were previously not fully open-source ^30^. Notably, emerging IAMs such as FRIDA ^13^, GCAM-Europe ^14^, NEMESIS-World, and WILIAM ^15^ have been, or are expected to be, released under open-source licenses.

Apart from the open-source licensing of IAMs and related models, modeling teams have recently started to produce standardized reporting procedures to harmonize model outputs, a critical step for facilitating effective result comparisons and enabling robust multi-model studies ^31– 33^. In parallel, to communicate effectively the model results, several user-friendly visualization platforms have emerged, standing out to stakeholders and policymakers. Key tools in this domain include the 1.5 ^◦^ C Scenario Explorer ^34^, the Global Hotspots Explorer ^16^, and the Sixth Assessment Report (AR6) Scenario Explorer ^35^. Moreover, various platforms, packages, and libraries have been developed to streamline the analysis, visualization, validation, and comparison of model outputs. Tools like pyam ^36^ assist in data analysis and figure creation, while others, such as nomenclature ^31^ and common-definitions ^37^ hold definitions and mappings to facilitate validation and processing of scenario data. Platforms like IAM2PARIS Validation Tool ^17^ vet the models’ outputs, while R packages like iamc ^38^ and gcamreport ^32^ standardize model outputs, enabling seamless multi-model comparisons. For visualizing model results, tools like witch-plot ^39^ offer specialized features tailored for modelers.

The development of all these tools facilitates multi-model comparison studies, which are crucial for improving the consistency and robustness of policy recommendations ^26, 40^. Open science strengthens the understanding and integration of models, while also streamlining data analysis by providing comprehensive documentation of model linkages ^25^. However, the implementation of open science practices varies widely across modeling teams and is often hindered by various factors, which can slow scientific progress, limit the reporting and reuse of research findings, and hamper the reliability of the representation of real-world complex systems ^8, 41– 45^.

To gain direct insights into the open science practices employed by IAMs’ modeling teams and the challenges they face, we surveyed nine modeling teams from the IAM COMPACT consortium ^46^. This Horizon Europe research project aims to support the assessment of global climate goals and the design of the next round of Nationally Determined Contributions and policy planning beyond 2030. With diverse multi-model analyses at its core, the project emphasizes the importance of using and standardizing open science practices among the modeling teams to ensure that model outputs are reliable, accessible, transparent, and reusable.

## Survey results

To identify the open science practices employed by various modeling teams, as well as the main challenges and barriers they face, we designed a survey ^47^. The survey was structured into three sections aligned with the traditional IAM modeling steps: model(s) development, calibration, and interconnection; model(s) output analysis and dissemination; and tools used throughout the process. Specifically, the first section focused on the documentation process, including model documentation, input harmonization, and potential multi-model connectivity. The second section examined the management of project outcomes, addressing output standardization, the vetting process, output storage, and results communication. Finally, the third section investigated the tools used or developed to support open science principles.

**Figure 1.**
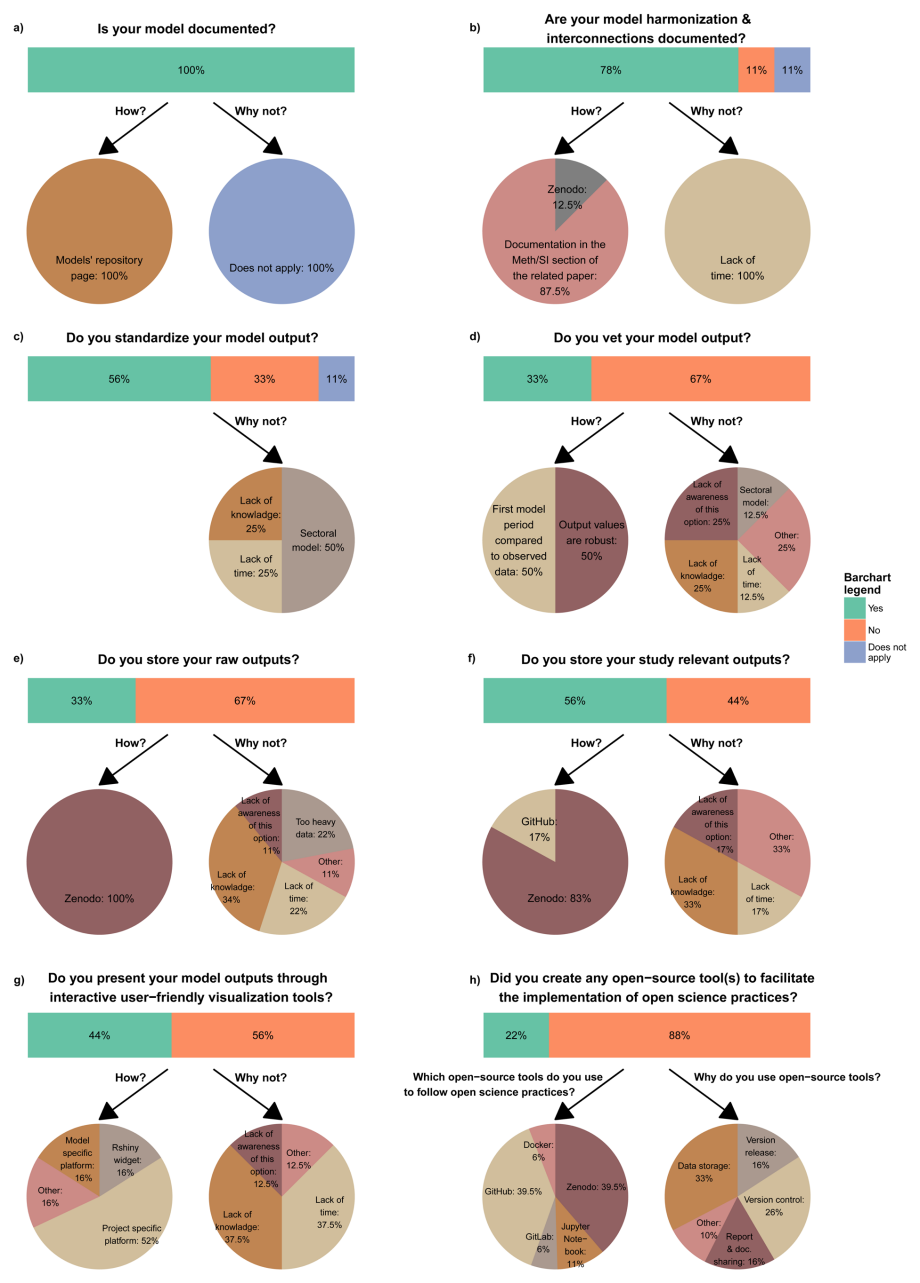
Summary statistics of responses to the primary survey questions.

As Figure 1 depicts, the consulted modeling teams document their models not only to ensure transparency and facilitate external use but also as a reliable internal reference when uncertainties arise. When models are interconnected, either through soft- or hard-linking, teams typically document the process, along with harmonization procedures, in the Methodology or Supplementary Information section of their papers. However, time constraints often prevent consistent documentation across teams ( Figure 1).

In terms of model output, not all teams adhere to the standardized reporting formats. IAM models generally recognize the advantages of using the IAMC time-series reporting template ^48^, which standardizes model outputs, facilitating comparisons across different models. However, sector-specific (e.g., energy) models, often linked to IAMs, tend to follow other reporting methods due to their more specialized and disaggregated results. Furthermore, not all modeling teams employ vetting procedures to validate their output. These procedures, designed to assess the accuracy of results, typically compare the first model period to observed data or check that key outputs, such as demand and supply, align correctly. However, these methods are unfamiliar to some teams and can be time-consuming, particularly when done manually rather than using automated tools ( Figure 1).

Lastly, modeling teams increasingly publish models’ raw output or relevant datasets on platforms like Zenodo ^18^, and more papers are now accompanied by the analysis code used in the study. However, due to heavy data weight, limited knowledge, or time constraints, this information is not always uploaded to a secure long-term location. To improve the communication of the results, many models are incorporating user-friendly interfaces, which, albeit requiring time to build and set up, make them more accessible and understandable for non-expert audiences ( Figure 1).

## Open Science Protocol

**Figure 2.**
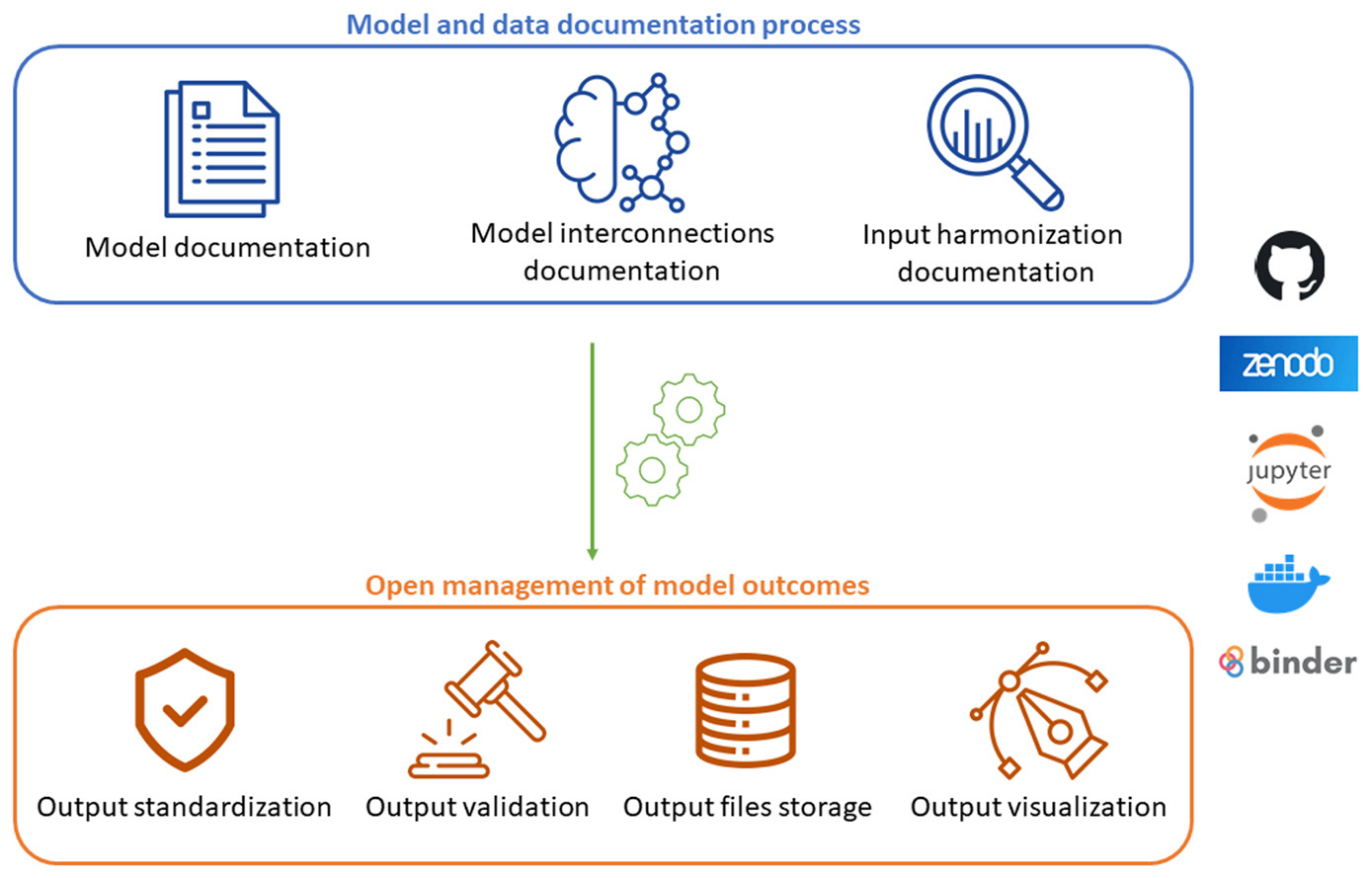
Scheme of the Open Science Protocol for the IAM community.

Based on the collected results, we propose an Open Science Protocol for the IAM community to encourage and support the adoption of open science practices ( Figure 2). The protocol structure aligns with the survey sections, with each stage designed to adhere to the FAIR and TRUST principles. It focuses on the key steps of IAM studies, offering detailed guidance and practical strategies to address the challenges identified through the survey. This protocol aims to ensure full transparency and thorough documentation of models, while making outcomes accessible, interoperable, reusable, reproducible, and reliable for a wide range of audiences, including both scientific and non-expert communities, serving as a dependable resource for informed decision-making.

The first stage of the protocol emphasizes the documentation and harmonization of models and data. It is crucial to ensure transparency in model assumptions, inputs, and outputs so that models developed by different teams can be interoperable and their results comparable. To achieve this, we recommend creating a dedicated platform for thorough documentation of each model with specific details about its versions. It is advisable to assign a Digital Object Identifier (DOI) to each model version release, ensuring that it is findable, accessible, reusable, and citable for everyone.

It is difficult to have a rule to report model interconnections since each case is unique. Thus, these details can be clearly outlined in the corresponding paper or case study, detailing how models interact, as well as the assumptions, inputs, outputs, and model versions used in the integration. If applicable, models’ inputs must be homogenized to have comparable results, a task that should be documented thoroughly in the corresponding paper. Additionally, publications should reference and cite the specific model versions used, including any modification details (if applicable), with links to the version repository if models are open-source. Modeling teams can leverage open-source platforms such as GitHub ^19^ for code hosting and version control, and Zenodo ^20^ for distributing model releases and, when needed, associated data.

The second stage of the protocol focuses on ensuring the quality and transparency of the outcomes. The first step is to standardize the outputs using the IAMC time-series data template ^48^, which facilitates cross-comparison of results between modeling teams and enables automated validation. While this procedure is specific to each model, it must be traceable, transparent, and reproducible. Automating this process is recommended, as it allows for the detection of errors or bugs over time ^32^.

To ensure consistency in the outputs, we suggest implementing a vetting procedure, ideally automated, using model-independent platforms like the IAM2PARIS Validation tool ^49^ or model-specific tools such as gcamreport ^32^. Through this process, the accuracy of the outcomes is assessed by verifying that paired outputs, such as supply and demand, are properly aligned, and by comparing the initial model period with observed data. With the automation of these processes, modeling teams can save time and effort, as well as ensure the clarity and replicability of the workflows.

To guarantee full transparency, accessibility, and reusability of results, it is advisable to upload the outputs to a platform under a DOI, such as Zenodo. However, as the survey indicates, it may not always be feasible to upload raw outputs due to their size. In such cases, the relevant study results and/or lighter standardized datasets should be made available. The shared outputs must be accompanied by detailed metadata documenting the data origin and the specific step-by-step data handling processes used to develop and reuse it. Assigning a DOI and detailing tailor-made metadata helps ensure that data is easily located and preserved over time, and facilitates its use in future research projects.

Finally, in order to efficiently share the findings with both the scientific community and the general public, the protocol suggests the dissemination of results through visualization tools that transform complex data outputs into comprehensible formats. Although creating these tools can be time-consuming, user-friendly visualizations enable clear exploration of the results and significantly enhance transparency, engaging policymakers and other stakeholders. Programming tools and platforms, such as dedicated websites, R Shiny apps ^21^, and visualization packages like pyam ^50^, can leverage the development of these interactive interfaces.

To facilitate the protocol application and boost the implementation of open science principles, we provide a checklist to modeling teams ^47^, available at Zenodo ^22^. This comprehensive list, structured in the same blocks as the protocol, serves as a useful tool to ensure that no steps are overlooked and acts as a reminder of best practices in open science.

## Discussion

Open science has developed into a major area of policy interest for academic institutions and a common approach for many researchers. The IAM community is rapidly adhering to these principles since IAM results hold particular significance for a wide range of stakeholders and policymakers. IAM models have traditionally faced criticism for their lack of transparency and the difficulty in fully testing their assumptions due to the complexity of the underlying code ^51^. This has led to perceptions of IAMs as “black box” models, diminishing their reliability in the eyes of some decision-makers ^41^. Embracing open science practices would greatly benefit the IAM community by demystifying these models and making their methods and procedures far more transparent and accessible. This is a crucial step in translating model insights into real-world policy actions.

As in other scientific communities, the adoption of open science principles faces several challenges, such as limited awareness, knowledge gaps, time constraints, limited financial resources, intellectual property concerns, and various cultural and social factors ^52– 54^. However, several scientific disciplines have established protocols, data repositories, and best practices (e.g., Nature Protocols ^23^, BioModels Database ^24^, and the Environmental Data Initiative ^25^), which help promote open science and ensure transparency. Similarly, projects like the Reproducibility Project Cancer Biology ^26^ and the Reproducibility Project Psychology ^55^ work to verify research reproducibility, thereby enhancing the credibility and reliability of scientific findings. While the IAM community has developed output standardization guidelines ^31^ and user-friendly visualization interfaces ^34, 35^, it still lacks a comprehensive protocol to guide the development of modeling studies.

To address this gap, this paper introduces a protocol for the entire IAM community that offers a structured framework for modeling teams and related projects. While it is built on the challenges faced by the IAM COMPACT modeling teams, the protocol aims to serve as a valuable resource for the broader community. It outlines best practices and recommended platforms and tools to streamline labor intensive tasks. By adopting this protocol, modeling teams can optimize time and resources while improving the transparency, accessibility, reliability, interpretability, and reusability of their results. With collective effort, the protocol can be continually updated and refined to better meet the evolving needs of modelers, ultimately advancing climate science and supporting the development of evidence-based policies.

## Ethics and consent

All survey respondents were members of the IAM COMPACT consortium and provided informed consent for the use of the information shared in this study. They were informed that their responses would be utilized for research purposes and included in this paper. The data were collected under the assurance of anonymity and were used exclusively to support the development of open science practices and protocols for integrated assessment models. This process was conducted in compliance with ethical approval from the Basque Centre for Climate Change (BC3) Management Committee. Respondents also retained the right to withdraw their participation at any point.

## Data Availability

The checklist and survey results are available in Zenodo ( https://zenodo.org/records/13970492) DOI: 10.5281/zenodo.13970492 ^47^ Data available under the terms of the Creative Commons Attribution 4.0 International license (CC-BY 4.0) ( https://creativecommons.org/licenses/by/4.0/).

## Footnotes

^1^ https://doaj.org/

^2^ https://data.worldbank.org/

^3^ https://www.who.int/data/gho/

^4^ https://unix.org/

^5^ https://git-scm.com/

^6^ https://research-and-innovation.ec.europa.eu/strategy/strategy-2020-2024/our-digital-future/open-science/european-open-science-cloud-eosc_en

^7^ https://openmod-initiative.org/

^8^ https://github.com/JGCRI/gcam-core

^9^ https://github.com/iiasa/message_ix

^10^ https://github.com/EnergySystemsModellingLab/MUSE_OS

^11^ https://github.com/remindmodel/remind

^12^ https://github.com/witch-team/witchmodel

^13^ https://github.com/metno/WorldTransFRIDA

^14^ https://github.com/bc3LC-GCAMEurope/gcam-core

^15^ https://github.com/LOCOMOTION-h2020

^16^ https://www.hotspots-explorer.org/

^17^ https://validation.i2am-paris.eu/

^18^ https://zenodo.org/

^19^ https://github.com/

^20^ https://zenodo.org/

^21^ https://shiny.posit.co/

^22^ https://doi.org/10.5281/zenodo.13970492

^23^ https://www.nature.com/nprot/

^24^ https://www.ebi.ac.uk/biomodels/

^25^ https://portal.edirepository.org/nis/home.jsp

^26^ https://www.cos.io/rpcb

## Software availability

Source code available from GitHub ( https://github.com/klau506/open_science_iams).

Archived software available from ( https://zenodo.org/records/13970492).

Data and archived software available under the terms of the Creative Commons Attribution 4.0 International license (CC-BY 4.0) ( https://creativecommons.org/licenses/by/4.0/).

Source code available under the terms of the Apache 2.0 license ( https://www.apache.org/licenses/).
