# Supplementary material for: Open Science Practices in Integrated Assessment Models
Source: Open Res Eur. 2025 Jun 16;5:12. Originally published 2025 Jan 20. [Version 2] doi: 10.12688/openreseurope.18824.2 (PMC12264473; doi:10.12688/openreseurope.18824.2)
Supplement: Peer review history

# Reviewer report: Reviewer response for version 2

**DOI:** 10.21956/openreseurope.22308.r55619

**Published:** 2025-07-23

**Authors:** Anne Fouilloux

**Affiliations:** 1 Simula Research Laboratory, Oslo, Norway

The authors have made substantial revisions that successfully address the major concerns raised in my initial review.

 The revised manuscript demonstrates significant improvement in scientific rigor and practical applicability: 

**- **The addition of a dedicated Methods section with clear survey design details;

 - The expansion from 9 to 13 responses is modest but I acknowledge the effort.

**- **The expanded background section now provides good coverage of FAIR and TRUST principles with extensive literature review and examples from related modeling communities.

**- **Table 1 provides concrete examples and recommended tools for each protocol step, transforming abstract recommendations into actionable guidance.

**- **Figure quality improvements and data format enhancements address reproducibility concerns.

 Minor Limitation: 

 - The sample size remains modest (n=13), though the authors have appropriately contextualized this within their target IAM modeling communities.

 The revised manuscript successfully addresses my concerns and provides a valuable resource for promoting open science practices in integrated assessment modeling.

If applicable, is the statistical analysis and its interpretation appropriate?

Yes

Is the study design appropriate and is the work technically sound?

Partly

Is the work clearly and accurately presented and does it engage with the current literature?

Partly

Are the conclusions drawn adequately supported by the results?

Partly

Are sufficient details of methods and analysis provided to allow replication by others?

Partly

Are all the source data and materials underlying the results available?

Yes

Reviewer Expertise:

FAIR and Open Science with a focus on Climate Science and more recently Climate Change Adaptation.

I confirm that I have read this submission and believe that I have an appropriate level of expertise to confirm that it is of an acceptable scientific standard.

# Reviewer report: Reviewer response for version 2

**DOI:** 10.21956/openreseurope.22308.r56055

**Published:** 2025-07-15

**Authors:** David Resnik

**Affiliations:** 1 National Institute of Environmental Health Sciences, Durham, USA

This study provides useful results for open sciences practice with integrated assessment modeling teams.  The methodology and survey are sound, the results are significant, and the paper is well written and well referenced.  The authors have mode appropriate revisions in response to the recommendations from the first round of review.  I recommended indexing.

If applicable, is the statistical analysis and its interpretation appropriate?

Yes

Is the study design appropriate and is the work technically sound?

Yes

Is the work clearly and accurately presented and does it engage with the current literature?

Yes

Are the conclusions drawn adequately supported by the results?

Yes

Are sufficient details of methods and analysis provided to allow replication by others?

Yes

Are all the source data and materials underlying the results available?

Yes

Reviewer Expertise:

research ethics

I confirm that I have read this submission and believe that I have an appropriate level of expertise to confirm that it is of an acceptable scientific standard.

# Reviewer report: Reviewer response for version 2

**DOI:** 10.21956/openreseurope.22308.r55620

**Published:** 2025-06-23

**Authors:** Marc-André Simard

**Affiliations:** 1 Université de Montréal, Québec,, Canada

I want to thank the authors for addressing all my comments and suggestions. I also appreciated all the improvements to the github repository, incluing the README file as well as the data format which both increase the paper’s openness and reproducibility.

 From an accessibility perspective (e.g. colors, text, etc.), I would make sure that all the figures are compatible with best accessibility practices to ensure that they can be viewed by a maximum number of people. Other than that, I believe that the paper is ready for indexing.

If applicable, is the statistical analysis and its interpretation appropriate?

Yes

Is the study design appropriate and is the work technically sound?

Yes

Is the work clearly and accurately presented and does it engage with the current literature?

Partly

Are the conclusions drawn adequately supported by the results?

Yes

Are sufficient details of methods and analysis provided to allow replication by others?

Partly

Are all the source data and materials underlying the results available?

Yes

Reviewer Expertise:

Information science, bibliometrics, science of science, open access, science policy

I confirm that I have read this submission and believe that I have an appropriate level of expertise to confirm that it is of an acceptable scientific standard.

# Reviewer report: Reviewer response for version 1

**DOI:** 10.21956/openreseurope.20371.r53079

**Published:** 2025-04-26

**Authors:** Anne Fouilloux

**Affiliations:** 1 Simula Research Laboratory, Oslo, Norway

Summary of the article:

 This paper presents an **Open Science Protocol** specifically tailored for the **Integrated Assessment Modeling (IAM)**community, based on a survey of IAM teams. The protocol is designed to improve the **transparency, accessibility, and reusability** of IAM models and outputs by aligning with the **FAIR** (Findable, Accessible, Interoperable, Reusable) and **TRUST** (Transparency, Responsibility, User focus, Sustainability, Technology) principles. The study highlights current challenges in implementing open science within IAMs—such as inconsistent documentation, limited vetting of outputs, and barriers to sharing data—and offers practical guidance to overcome these. By fostering better communication and usability of models, especially for policymakers, the protocol aims to enhance the **credibility and impact** of IAM-based climate assessments.

 Details related to the questions above (where I answered partly):

 The paper acknowledges the importance of the FAIR (Findability, Accessibility, Interoperability, and Reusability) and TRUST (Transparency, Responsibility, User focus, Sustainability, and Technology) principles but does not fully explore their practical implementation within Integrated Assessment Models (IAMs). A deeper engagement with recent literature and case studies specific to IAMs and these principles would strengthen both the theoretical foundation and practical relevance.

 While the survey design is appropriate for gathering qualitative insights, the methodology section lacks sufficient detail. Key elements such as the full survey instrument, sample size, and selection process are missing, which limits transparency and hampers replicability. Providing this information, along with a clearer description of how responses were analyzed, would enhance the robustness of the conclusions.

 The protocol presented is promising, with clearly outlined steps. However, it would benefit from further elaboration, particularly regarding practical challenges encountered, strategies for adaptation across IAM contexts, and concrete examples of successful implementation. Additional data—such as more detailed documentation, case studies, or checklists—would improve the transparency and reusability of the work.

 Although the interpretation of survey results is reasonable, it relies heavily on qualitative insights. Incorporating statistical analysis or quantitative data could provide stronger support for the findings. The conclusions align with the presented results but could be made more compelling through the inclusion of real-world applications that demonstrate the protocol’s effectiveness and impact.

 Other information related to data/code availability and figure:

 - The GitHub repository: https://github.com/klau506/open_science_iams does not detail how to use the R code and with which data.

 - The data is deposited in Zenodo as xlsx file which is not an open format. csv file (text file) would be better for reuse.

 - Figure 1 is unreadable on my laptop (too small). 

 - The survey results file (available at https://zenodo.org/records/13970492) contains 9 entries, suggesting that fewer than 10 individuals responded to the survey.

 Recommendation:

 There are a few areas where additional details and refinement could help to strengthen the scientific rigor of your paper. Below are some specific suggestions:

 1. **Strengthen the Theoretical Framework**

 It would be beneficial to gain a deeper understanding of how the FAIR and TRUST  principles can be practically applied to IAMs (in detail because in the paper, the 15 FAIR principles are not addressed with sufficient details). This will help better align the theoretical foundation of your protocol with current best practices in Open Science and ensure its relevance to the IAM community.

 2. **Enhance Methodological Transparency**

 The methodology section could be improved by providing more **detailed information about the survey design**. For instance, explain why the **sample size** and the **selection process** of survey participants were sufficient for this work.

 3. **Improve the GitHub Repository**

 The **GitHub repository** could be enhanced by providing clear **documentation** on how to use the R code and apply it to the provided data. Additionally, I suggest switching the data format from **XLSX** to an **open format like CSV** to facilitate easier reuse and interoperability with other tools.

 4. **Enhance Visual Accessibility**

**Figure 1 i**s not easily readable on standard devices. I recommend increasing the **resolution** and **size** of the figures to improve accessibility, ensuring that they are clearly visible to a broader audience.

 5. **Increase Survey Sample Size**

 Given that the survey currently includes fewer than 10 responses, I suggest reaching out to more IAM teams to **increase the sample size**. A larger sample would improve the **representativeness** of the findings. It would also be helpful to acknowledge the potential limitations posed by the small sample size and how these might affect the interpretation of your results.

If applicable, is the statistical analysis and its interpretation appropriate?

Yes

Is the study design appropriate and is the work technically sound?

Partly

Is the work clearly and accurately presented and does it engage with the current literature?

Partly

Are the conclusions drawn adequately supported by the results?

Partly

Are sufficient details of methods and analysis provided to allow replication by others?

Partly

Are all the source data and materials underlying the results available?

Yes

Reviewer Expertise:

FAIR and Open Science with a focus on Climate Science and more recently Climate Change Adaptation.

I confirm that I have read this submission and believe that I have an appropriate level of expertise to confirm that it is of an acceptable scientific standard, however I have significant reservations, as outlined above.

# Reviewer report: Reviewer response for version 1

**DOI:** 10.21956/openreseurope.20371.r50728

**Published:** 2025-02-26

**Authors:** Marc-André Simard

**Affiliations:** 1 Université de Montréal, Québec,, Canada

**Overall:**

 Using survey data, this manuscript investigates open science practices (including barriers and challenges) in integrated assessment modeling (IAM) teams. Furthermore, this manuscript proposes an open science protocol to address some key challenges and barriers (such as data weight, lack of time, awareness or knowledge, etc.) in the use of open science within integrated assessment modeling. The protocol is built around validated principles such as FAIR and TRUST. The authors provide sufficient context and information (e.g. packages, checklist, softwares, plaforms, etc.) for their model to be adaptable and usable in a wide range of contexts.

 I do believe that this manuscript would make a great addition to the body of literature on open science, but I believe that there are some things that could be addressed before it is ready to be indexed.  

 While I understand that the intention of the authors is most likely to keep the introduction/review of literature brief while attempting to cover the massive landscape of OS, it can sometimes feel like some of these issues are tackled in a slightly superficial manner. For instance, a few of the paragraphs could be more fleshed out and expanded into proper sections (e.g. open data, benefits of OS) which could cover some recent empirical research results/literature on these topics.

 Further, while I also greatly appreciate the transparency and the sharing of the data, codes, protocols, etc., I think the manuscript would also benefit to have a dedicated methodology section; most of the information is already available in the “Survey Results” section. I would just separate it into two sections and flesh it out a little bit more if/when needed with some descriptive information about the methods, participants, etc.  

 Some more specific comments:

**Introduction/review of literature: **

- Regarding OS initiatives in the second paragraph: I would clarify a couple of things regarding the US’ policies. Many US OS policies (including the NIH’s) are normally called “public access policies” because they have slightly different criteria from OA policies. The Nelson Memo (Refer 1) has also sparked a new generation of OS policies in the US; the NIH’s latest policy was recently released. In short, I would cover the Nelson Memo and then would give some of the newest policies as examples instead of the NIH’s previous 2021 policy.
- In the third paragraph (resources) I would also mention an OA repository, given how important they have been. OpenAIRE/CERN’s Zenodo or PubMed Central would be a good example.

**OS practices in IAMs: **

- As someone who is not too familiar with IAMs, I would have appreciated a sentence or two on their background. A Google search helped me catch up quickly, but the additional information wouldn’t hurt.

**Results: **

- The figures are beautifully done, but the text in the pie charts (Figure 1) is tiny and can be hard to read on a laptop. I am also not sure if the colours are on par with accessibility standards (e.g. colours, black and white for print, etc.). I would verify that if it hasn’t already been done.

If applicable, is the statistical analysis and its interpretation appropriate?

Yes

Is the study design appropriate and is the work technically sound?

Yes

Is the work clearly and accurately presented and does it engage with the current literature?

Partly

Are the conclusions drawn adequately supported by the results?

Yes

Are sufficient details of methods and analysis provided to allow replication by others?

Partly

Are all the source data and materials underlying the results available?

Yes

Reviewer Expertise:

Information science, bibliometrics, science of science, open access, science policy

I confirm that I have read this submission and believe that I have an appropriate level of expertise to confirm that it is of an acceptable scientific standard, however I have significant reservations, as outlined above.

## References

[1] : MEMORANDUM FOR THE HEADS OF EXECUTIVE DEPARTMENTS AND AGENCIES.2022 https://bidenwhitehouse.archives.gov/wp-content/uploads/2022/08/08-2022-OSTP-Public-Access-Memo.pdf;
